# Diarrhoeal diseases in Soweto, South Africa, 2020: a cross-sectional community survey

**DOI:** 10.1186/s12889-021-11470-9

**Published:** 2021-07-20

**Authors:** Siobhan L. Johnstone, Nicola A. Page, Juno Thomas, Shabir A. Madhi, Portia Mutevedzi, Nellie Myburgh, Carlos Herrera, Michelle J. Groome

**Affiliations:** 1 Center for Enteric Diseases, National Institute for Communicable Diseases, Johannesburg, South Africa; 2 School of Public Health, Faculty of Health Sciences, University of the Witwatersrand, Johannesburg, South Africa; 3 Department of Medical Virology, Faculty of Health Sciences, University of Pretoria, Private Bag X323, Arcadia, 0007 South Africa; 4 South African Medical Research Council: Vaccines and Infectious Diseases Analytics Research Unit, Faculty of Health Sciences, University of the Witwatersrand, Johannesburg, South Africa

**Keywords:** Diarrhoea, Community, Handwashing, Adults, Children, ORS

## Abstract

**Background:**

In South Africa, there are limited data on the burden of diarrhoea at a community level, specifically in older children and adults. This community survey estimated rates of and factors associated with diarrhoea across all ages and determined the proportion of cases presenting to healthcare facilities.

**Methods:**

Households were enrolled from an existing urban health and demographic surveillance site. A household representative was interviewed to determine associated factors and occurrence of diarrhoea in the household, for all household members, in the past 2 weeks (including symptoms and health seeking behaviour). Diarrhoeal rate of any severity was calculated for < 5 years, 5–15 years and > 15 years age groups. Factors associated with diarrhoea and health seeking behaviour were investigated using binomial logistic regression.

**Results:**

Diarrhoeal rate among respondents (2.5 episodes/person-year (95% CI, 1.8–3.5)) was significantly higher than for other household members (1.0 episodes/person-year (95% CI, 0.8–1.4); IRR = 2.4 (95% CI, 1.5–3.7) *p* < 0.001). Diarrhoeal rates were similar between age groups, however younger children (< 5 years) were more likely to present to healthcare facilities than adults (OR = 5.9 (95% CI, 1.1–31.4), *p* = 0.039). Oral rehydration solution was used in 44.8% of cases. Having a child between 5 and 15 years in the household was associated with diarrhoea (OR = 2.3 (95% CI, 1.3–3.9), *p* = 0.003) and, while 26.4% of cases sought healthcare, only 4.6% were hospitalised and only 3.4% of cases had a stool specimen collected. While the majority of cases were mild, 13.8% of cases felt they required healthcare but were unable to access it.

**Conclusion:**

Diarrhoeal rate was high across all age groups in this community; however, older children and adults were less likely to present to healthcare, and are therefore underrepresented through facility-based clinical surveillance. Current diarrhoeal surveillance represents a fraction of the overall cases occurring in the community.

**Supplementary Information:**

The online version contains supplementary material available at 10.1186/s12889-021-11470-9.

## Summary of article’s main point

This study estimated diarrhoeal rate at a community level across all age groups in an urban township in South Africa. Factors associated with diarrhoea in the household, the proportion of cases presenting to healthcare, and factors associated with health seeking were determined.

## Background

Although progress has been made towards improving water and sanitation globally, diarrhoea has remained in the top 10 causes of mortality and morbidity among all ages [1, 2]. In 2016, diarrhoea was the eighth leading cause of death among all ages (1,655,944 deaths) and the fifth leading cause of death among children under 5 years of age (446,000 deaths) [1]. Nutritional wasting in young children, unsafe water and poor sanitation were the leading factors associated with diarrhoeal morbidity and mortality [1, 3]. Overall, global childhood diarrhoeal diseases have decreased in the past 10 years, largely due to improved maternal education, access to rotavirus vaccination, and improvement of child growth due to better nutrition [1]. However, this decrease has not been uniform across age groups or across settings. Focussed attention on older populations (where there are large knowledge gaps in terms of aetiology and epidemiology) [1, 4] and low-income settings (which still bear the brunt of the burden of disease) [1] is required. Estimates show that the vast majority of global diarrhoeal deaths occur in south Asia and sub-Saharan Africa [1, 5].

Diarrhoeal pathogens are commonly transmitted through the faecal-oral route, due to poor hygiene and sanitation [6], and through ingestion of contaminated food and water, aided by poor food safety practices [7]. Diarrhoeal morbidity is therefore largely preventable through improved access to safe water and sanitation and by ensuring communities are well educated on good handwashing and safe food preparation practices. The WHO has defined five keys to safer food in order to simplify the messaging behind food safety [8]. Diarrhoeal deaths are also largely preventable if dehydration is properly managed [9]. Dehydration can be prevented through a simple, homemade, sugar and salt solution or oral rehydration solution (ORS) as recommended by the WHO [10, 11]. ORS is estimated to reduce diarrhoeal mortality by up to 93% at a healthcare level, although less is known about its impact and use at a community level [9]. Many of the interventions required to reduce diarrhoeal mortality and morbidity are relatively simple and can be addressed through community education. Diarrhoea is therefore one of the most tangible targets for reducing mortality and morbidity from preventable diseases.

Current diarrhoeal surveillance studies being conducted at several hospitals throughout South Africa enrol patients of all ages hospitalised for acute, moderate to severe diarrhoea. However, cases enrolled in these studies represent only a fraction of diarrhoea in the community and are biased towards people with better access to healthcare services. It is also important to understand the healthcare utilization patterns in the community when interpreting the data from hospital surveillance studies. This cross-sectional, questionnaire-based, community survey was undertaken to estimate the rate of and factors associated with diarrhoea in the community across all age groups in Soweto, South Africa and to determine the proportion of cases presenting to healthcare facilities. Secondary objectives were to investigate ORS knowledge and barriers to accessing healthcare in this community.

## Methods

### Study area and population

Soweto is a densely populated, urban township in Johannesburg, South Africa with an estimated population of 1.3 million people in 355,331 households (2011 census) [12]. According to the most recent census, 97% of residents have access to potable water provided by the municipality, with 55% having access to piped water inside the dwelling. Ninety two percent of residents use flush toilets [12]. Unemployment is high with 19% of households not receiving any set income and a dependency ratio of 40.8 [12]. The average household size is 3.4 people [12] with an average household income of R6500 ($455) per month [13]. Soweto is served by Chris Hani Baragwanath Academic Hospital, a large, secondary-tertiary care hospital, Bheki Mlangeni District Hospital, and several public clinics and private practitioners [13].

The Soweto health and demographic surveillance site (HDSS) was established in 2017 as part of the Child Health and Mortality Prevention Surveillance (CHAMPS) network [14] and currently tracks individuals from 20,778 households in eight clusters in Soweto through biannual data collection rounds. This diarrhoeal diseases survey used the Soweto HDSS as a sampling frame.

### Sampling methods and data collection

Probability proportional to size sampling was used to select four of the eight Soweto clusters (due to limited resources and relative size of the clusters) from which households were then randomly sampled. Soweto HDSS data were used to verify that clusters were not significantly different in terms of socioeconomic status.

To obtain a representative sample of each of the four clusters with a 5% precision, 95% confidence level, using an estimated 2-week diarrhoeal prevalence of 6% [15] (amongst all ages), a survey size of 84 households was required per cluster with a total of 336 households in all four clusters. Non-response rate was estimated at 30% hence 500 households were selected (125 in each of the four clusters). Fieldworkers visited the selected households, explained the study to an adult (≥18 years old) representative of the household, and obtained written informed consent. A questionnaire was administered in the preferred language of the respondent. The questionnaire included sections on handwashing practices; eating and food preparation practices (including where food is purchased, and how it is stored and prepared); water (source of drinking water, water treatment and storage); sanitation; and ORS use and knowledge. Respondents were also asked if any members of the household had experienced a diarrhoeal episode (defined as ≥3 loose or liquid stools in 24 h for any duration) in the past 2 weeks. Further questions on symptoms (as per other diarrhoeal community studies [16]) and health seeking behaviour were included for households with a reported diarrhoeal episode. Questions on diarrhoeal episodes were included for all members of the household in order to avoid selection bias resulting from limiting the survey to include only individuals found to be at home during the day. Households not available on the first visit were visited on a second occasion and considered a non-response if not available at either visit. Data collection was completed over a one-month period in February 2020.

### Statistical analysis

The cluster design of the study was accounted for by specifying data as three-level, complex survey data (cluster, household and individuals within the household as the three levels). Demographic and socioeconomic information (including dwelling type, structure main material, home ownership, power source used for cooking, and toilet type) was obtained for enrolled households from HDSS data, using respondent name, surname and age, before being de-identified for the purposes of the analysis. The International Wealth Index (IWI) [17] was used as a composite measure of material wealth for each household. This measure combines assets, housing floor material, toilet facility, number of rooms, access to electricity and water source.

The number of individuals living with the respondent (as reported by the respondent) was used as the denominator for the two-week diarrhoeal rate. Respondents only answered questions pertaining to their household; individuals living in a separate dwelling on the same property were excluded as it was unlikely that the respondent would have been able to accurately answer questions pertaining to these individuals. Handwashing practices were considered adequate if the respondent reported always washing their hands with both soap and water (as opposed to water only) at critical times, including before eating and preparing food and feeding children, as well as after using the toilet and changing children’s diapers.

Diarrhoeal rate was calculated as episodes per person-year (PY) using events per person over the 2-week period. Confidence intervals (95%) for diarrhoeal rates were calculated using the Poisson distribution. Incidence rate ratios (IRR) were calculated to compare the diarrhoeal rates among strata (including age groups; type of diarrhoea; and episodes reported for respondents versus other household members). Factors associated with at least one diarrhoeal episode being reported for a household were investigated using binomial logistic regression modelling. Health seeking (defined as seeking healthcare at a clinic, hospital, general practitioner or pharmacy) was investigated for reported diarrhoeal episodes, using binomial logistic regression modelling. Multivariate analysis included all variables significant at *p*-value< 0.15 in the univariate analysis and used backwards, stepwise selection (using likelihood-ratio test) to determine which variables to retain in the multivariate model. Households where the respondent could not be matched to the HDSS data (as they may have relocated between the most recent HDSS round and the current survey) were excluded from the multivariate analysis. Factors associated with ORS knowledge were investigated using X^2^-test for categorical variables and t-test for continuous variables. Non-response rates were compared to ensure that there were no significant differences between clusters. Stata software (version 14) was used for all analyses.

### Ethical considerations

This study was approved by the Human Research Ethics Committee (Medical) of the University of the Witwatersrand (approval number: M190663) and the CHAMPS Soweto HDSS Community Advisory Board.

## Results

### Enrolled households and respondents

**Fig. 1:**
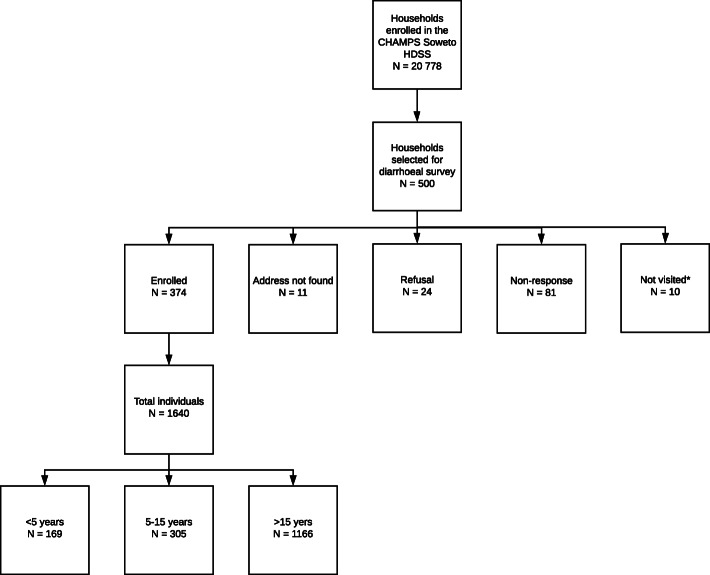
Enrolment flow diagram. *10 households were not visited due to strike action which prevented fieldwork for several days. Despite this, the required sample size was reached

A total of 374 households comprising 1640 individuals (77.2% of which were reported by proxy), were enrolled (Fig. 1). Respondents were majority female (67.4%) with a median age of 45 years (IQR: 24–59). A total of 355 (94.9%) respondents could be matched to the CHAMPS HDSS data.

### Diarrhoeal rate

Of the 374 households surveyed, 78 (20.9%) reported at least one diarrhoeal episode in the past 2 weeks. Seventy-one (91.0%) of these had a single episode per household, six (7.7%) had two episodes, and one (1.3%) had four episodes. Hence, a total of 87 diarrhoeal episodes were reported, 36 (41.4%) of which were self-reported by the respondent and 51 (58.6%) were reported on behalf of someone else in the household.

The overall 2-week diarrhoeal prevalence for the surveyed population was 5.3% which translates to a rate of 1.4 episodes/PY (95% CI, 1.1–1.7) (Table 1). Acute diarrhoea (< 14 days) was common (1.3 episodes/PY (95% CI, 1.0–1.6)) while persistent diarrhoea was rare (0.1 episodes/PY (95% CI, 0.0–0.2)). Reported 2-week prevalence for respondents was 9.6% (rate of 2.5 episodes/PY (95% CI, 1.8–3.5)) which was significantly higher than reported for other household members (2-week prevalence of 4.0% and rate of 1.0 episodes/PY (95% CI, 0.8–1.4)) as shown by the IRR of 2.4 (95% CI, 1.5–3.7, *p* < 0.001). Rates between age groups were similar (1.1 episodes/PY (95% CI, 0.4–2.2) in < 5 years; 1.3 episodes/PY (95% CI, 0.8–2.2) in 5–15 years; 1.4 episodes/PY (95% CI, 1.1–1.8) in > 15 years).

**Table 1:** Rate of diarrhoeal disease reported for different groups

	Events (N)	Denominator (number of people)	2-week prevalence (%)	Person-years	Rate (episodes per person-year (95% CI))
Overall	87	1640	5.3	62.9	1.4 (1.1–1.7)
Acute (< 14 days)	81	1640	4.9	62.9	1.3 (1.0–1.6)
Persistent (≥14 days)	6	1640	0.4	62.9	0.1 (0.0–0.2)
Respondents	36	374	9.6	14.3	2.5 (1.8–3.5)
Other household members	51	1266	4.0	48.6	1.0 (0.8–1.4)
< 5 years	7	169	4.1	6.5	1.1 (0.4–2.2)
5–15 years	16	310	5.2	11.9	1.3 (0.8–2.2)
> 15 years	64	1171	5.5	44.9	1.4 (1.1–1.8)

### Factors associated with diarrhoeal episodes

Multivariate analysis found that having children aged 5–15 years in the household resulted in higher odds of having had a diarrhoeal episode in the household in the last 2 weeks (OR = 2.3 (95% CI, 1.3–3.9), *p* = 0.002), (Table 2). Inadequate handwashing (OR = 1.7 (95% CI, 1.0–3.0), *p* = 0.064) and having a flush toilet in the house compared to a flush toilet in the yard (OR = 1.7 (95% CI, 1.0–3.0), *p* = 0.057) were also associated with increased diarrhoeal episodes in the household; however, these did not reach statistical significance at the multivariate level. Number of people in the household, dwelling type, IWI, eating habits, where food was purchased (formal or informal traders) and stored (availability of cold storage), knowledge on separation of raw and cooked food, and water treatment, interruptions and storage were not associated with increased diarrhoeal episodes.

**Table 2:** Factors associated with occurrence of diarrhoeal episodes in the household in the preceding two weeks

	Diarrhoeal episode in the household n/N (%) ^c^	Univariate ^d^		Multivariate ^d, e^	
		OR (95% CI)	***p***-value	OR (95% CI)	***p***-value
Dwelling type					
Formal	56/74 (75.7)	Referent	–	–	–
Informal	18/74 (24.3)	1.6 (0.9–3.0)	0.135		
Structure main material					
Brick	66/74 (89.2)	Referent	–	–	–
Metal sheets	8/74 (10.8)	1.1 (0.5–2.6)	0.765		
Home ownership					
Owned by residents	38/74 (51.4)	Referent	–	–	–
Rented	27/74 (36.5)	1.7 (1.0–3.0)	0.071		
Government issued	9/74 (12.2)	0.9 (0.4–1.9)	0.718		
Power source for cooking					
Electricity	74/74 (100.0)	Omitted		–	–
Paraffin	0/74 (0)				
Toilet type					
Flush toilet in yard	22/74 (29.7)	Referent	**–**	Referent	–
Flush toilet in house	52/74 (70.3)	1.8 (1.0–3.1)	**0.038**	1.7 (1.0–3.0)	0.057
Ventilated pit latrine	0/74 (0)	–	–	–	–
International Wealth Index					
Median (IQR)	85.6 (79.1–92.1)	1.0 (1.0–1.0)	0.827	–	–
Number of people living in the household					
Median (IQR)	5 (3-7)	1.1 (1.0–1.2)	**0.004**	–	–
Children < 5 years in the household					
No	42/78 (53.9)	Referent	**–**	–	–
Yes	36/78 (46.1)	2.0 (1.2–3.4)	**0.008**		
Children between 5 and 15 years in the household					
No	29/78 (37.2)	Referent	**–**	Referent	–
Yes	49/78 (62.8)	2.3 (1.4–3.9)	**0.002**	2.3 (1.3–3.9)	**0.002**
Handwashing ^a^					
Adequate	24/78 (30.8)	Referent	**–**	Referent	–
Inadequate	54/78 (69.2)	1.8 (1.0–3.0)	**0.039**	1.7 (1.0–3.0)	0.064
Consume fresh fruit and vegetables ^b^					
Never	3/77 (3.9)	Referent	–	–	–
Occasionally	24/77 (31.2)	0.5 (0.1–2.1)	0.307		
Often	50/77 (64.9)	0.4 (0.1–1.8)	0.236		
Consume meat ^b^					
Never	3/78 (3.9)	Referent	–	–	–
Occasionally	28/78 (35.9)	1.4 (0.4–5.2)	0.617		
Often	47/78 (60.3)	1.2 (0.3–4.2)	0.815		
Consume dairy ^b^					
Never	10/77 (13.0)	Referent	–	–	–
Occasionally	28/77 (36.4)	1.2 (0.5–2.8)	0.710		
Often	39/77 (50.7)	0.8 (0.3–1.7)	0.520		
Consume eggs ^b^					
Never	16/77 (20.8)	Referent	–	–	–
Occasionally	26/77 (33.8)	0.7 (0.4–1.5)	0.420		
Often	35/77 (45.5)	0.7 (0.3–1.3)	0.224		
Consume ready-to-eat meat products ^b^					
Never	21/77 (27.3)	Referent	–	–	–
Occasionally	28/77 (36.4)	1.4 (0.7–2.7)	0.341		
Often	28/77 (36.4)	1.4 (0.7–2.6)	0.391		
Consume take-aways ^b^					
Never	28/77 (36.4)	Referent	–	–	–
Occasionally	39/77 (50.7)	1.3 (0.8–2.3)	0.313		
Often	10/77 (13.0)	1.8 (0.8–4.1)	0.182		
Eat at restaurants ^b^					
Never	48/78 (61.5)	Referent	–	–	–
Occasionally	28/78 (35.9)	1.0 (0.6–1.7)	0.937		
Often	2/78 (2.6)	1.3 (0.2–6.5)	0.771		
Purchase meat					
Informal	2/78 (2.6)	Referent		–	–
Commercial	76/78 (97.4)	1.9 (0.4–8.5)	0.408		
Purchase fruit and vegetables					
Informal	39/76 (51.3)	Referent	–	–	–
Commercial	37/76 (48.7)	1.2 (0.7–2.0)	0.451		
Fridge/freezer storage					
No	2/78 (2.6)	Referent	–	–	–
Yes	76/78 (97.4)	1.2 (0.3–5.7)	0.822		
Separate raw and cooked food					
No	1/78 (1.3)	Referent	–	–	–
Yes	77/78 (98.7)	2.8 (0.4–22.0)	0.336		
Treat drinking water					
No	67/78 (85.9)	Referent	–	–	–
Yes	11/78 (14.1)	1.6 (0.7–3.5)	0.251		
Water storage					
None (straight from tap)	47/78 (60.3)	Referent	–	–	–
Closed container	29/78 (37.2)	1.1 (0.7–1.9)	0.658		
Open container	2/78 (2.6)	1.6 (0.3–8.3)	0.600		
Interruptions to water supply in the past 2 weeks					
No	69/76 (90.8)	Referent	–	–	–
Yes	7/76 (9.2)	0.7 (0.3–1.7)	0.393		

^a^ Adequate defined as washing with soap and water at critical times (after using the toilet or changing diapers, and before preparing food, eating or feeding young children); ^b^ Occasionally defined as once/twice per week, and often defined as every day or every second day. ^c^ Denominator differs due to missing responses for some households (maximum of 78 households that experienced at least one episode of diarrhoea in the past two weeks); ^d^ Univariate and multivariate analysis using only the 355 households that could be matched to HDSS data. ^e^ The following variables were assessed in the multivariate model: number of people living in the household, dwelling type, home ownership, toilet type, children < 5 years in the household, children between 5 and 15 years in the household and handwashing. The following variables were retained in the model: toilet type, children between 5 and 15 years in the household and handwashing

### Symptoms and health seeking behaviour

The median age for those with diarrhoea was 30 years (IQR: 13–53). Episodes lasted between a few hours to 28 days with a median of 2 days (IQR: 2–5). Abdominal cramps were the most commonly reported symptom (59.8%) followed by headache (31.0%), loss of appetite (31.0%) and fever (31.0%), (Table 3). Some cases (14.9%) experienced no symptoms additional to the diarrhoea.

**Table 3:** Frequency of concurrently reported symptoms amongst household members with diarrhoeal episodes and factors associated with seeking healthcare

	Frequency of symptoms (%)	Factors associated with seeking healthcare	
		Univariate analysis ^a^	
		OR (95% CI)	*p*-value
Abdominal cramps	52 (59.8)	1.1 (0.4–2.9)	0.858
Headache	27 (31.0)	0.7 (0.2–2.1)	0.523
Loss of appetite	27 (31.0)	0.9 (0.3–2.7)	0.908
Fever	27 (31.0)	0.7 (0.2–2.1)	0.523
Myalgia	24 (27.6)	2.7 (1.0–7.4)	0.056
Respiratory symptoms ^b^	17 (19.5)	0.8 (0.2–2.8)	0.738
Nausea	15 (17.2)	2.1 (0.7–6.8)	0.208
Watery stool	14 (16.1)	0.7 (0.2–2.8)	0.625
Vomiting	13 (14.9)	1.3 (0.4–4.6)	0.722
Blood in stool	4 (4.6)	2.9 (0.4–21.9)	0.301
Age group (years)			
< 5	–	3.6 (0.7–17.8)	0.115
5–15		0.4 (0.1–1.9)	0.239
> 15		Referent	–
Female	–	0.8 (0.3–2.3)	0.634
International Wealth Index	–		
		1.0 (1.0–1.1)	0.194

^a^ The following variables were assessed and retained in the multivariate model: myalgia (OR = 3.4 (95% CI, 1.2–10.2), *p* = 0.027) and age group (OR = 5.9 (95% CI, 1.1–31.4), *p* = 0.039, for children < 5 years compared to those > 15 years). ^b^ Respiratory symptoms included cough, coryza and shortness of breath

Twenty-three of the 87 people with diarrhoea (26.4%) sought healthcare (Supplementary Figure S1). Fourteen (16.1%) visited a public clinic, while five (5.7%) visited a pharmacy and four (4.6%) were admitted to hospital. The admitted cases included a 4-month-old infant, two elderly adults (aged 65 and 77 years old) and a 23-year-old adult with dysentery, myalgia, abdominal cramps, fever, nausea and vomiting. Fifty-one (58.6%) cases did not seek healthcare as they felt their illness was mild, while 12 (13.8%) cases felt it was necessary but were unable to access healthcare. Reasons for not being able to access healthcare included personal issues (6/12, 50.0%) including not being able to take time off from work or home duties, and not having access to transport; as well as issues with the healthcare system (6/12, 50.0%), including being previously ill-treated at public clinics, long waiting times at the public clinic, and the public clinic being closed. In the multivariate analysis, children < 5 years and those with myalgia were significantly more likely to seek healthcare for diarrhoea compared with older children and adults and those without myalgia (OR = 5.9 (95% CI, 1.1–31.4), *p* = 0.039; OR = 3.4 (95% CI, 1.2–10.2), *p* = 0.027 respectively), (Table 3). The six cases that reported blood in the stool or prolonged symptoms all felt they required healthcare, although only three (50.0%) were able to access it. Stool specimens were collected in only three of the 87 cases (3.4%) or 16.7% of those that visited a public clinic or hospital (3/18) for their illness.

Only 44.8% (*n* = 39) of cases used ORS during the episode. Knowledge of ORS was poor in the surveyed population with only 51.0% (*n* = 192) of respondents having some knowledge of ORS (knew the recipe or were able to name the ingredients) and only 17.9% (*n* = 67) able to give the correct recipe. Females (*p* < 0.001), respondents with a child < 5 years old in the household (*p* = 0.010) or children between the age of 5 to 15 years in the household (*p* = 0.002) were significantly more likely to have some knowledge of ORS compared with males and respondents without children in the household (Supplementary Table S1).

## Discussion

The current perspective of diarrhoeal diseases in many low- and middle- income countries, including South Africa, is based largely on healthcare-level data focused on children under the age of 5 years [18, 19]. This study adds to the limited understanding of diarrhoeal diseases in all ages at a community level and assists in interpreting how representative healthcare and laboratory-level data are of these cases. This survey found a diarrhoeal rate of 2.5 episodes/PY (95% CI, 1.8–3.5) for respondents and 1.0 episodes/PY (95% CI, 0.8–1.4) for other household members (as reported by respondent as a proxy). Since respondents should not be different to other household members in terms of risk factors for diarrhoeal diseases, it is unlikely that this is a true difference and may be due to reporting bias (in which respondents underestimated episodes experienced by other household members) or, less likely, recall bias (in which respondents overestimate episodes experienced themselves). Since other studies using similar methods [3, 20] did not differentiate self-reported episodes to episodes reported on behalf of other household members, this difference cannot be compared to literature and requires further investigation. Both the self-reported rate and the rate for other household members were higher than those reported in high-income countries [15, 21]. This is expected, due to poorer living conditions and a higher burden of underlying conditions, including HIV and malnutrition, associated with increased diarrhoeal morbidity in our setting. The rates reported here are lower than those reported from other African countries, such as a household survey in Zambia which reported a rate of 1.7 episodes/PY for persistent diarrhoea in adults [22], and a household study in Ethiopia which found a diarrhoeal rate of 3.8 episodes/PY for children < 5 years of age [23]. Our reported prevalence for children < 5 years was similar to that previously reported in a community-based study from the same setting (4.0%) [13]. Rates for other household members are in keeping with the GBD estimates of 1.0 episodes/PY (95% CI, 1.0–1.1) for sub-Saharan Africa [1]. Since community-level data amongst all ages in sub-Saharan Africa are limited, it is possible that the higher self-reported estimates are accurate, and other estimates (based on healthcare-level data) are an underestimation. Unlike reports from studies in high income settings [15, 21], our survey found no significant difference between rates for different age groups. We did however find that healthcare was most likely to be sought for children < 5 years of age. This highlights that diarrhoeal cases in children < 5 years are seen disproportionally at a healthcare level in this community since many older children and adults do not seek healthcare for diarrhoeal episodes. The economic effects of diarrhoeal disease in the community therefore extend beyond healthcare system costs, and includes the reduction of economically active days for individuals of working age, causing social disturbance and lost economic opportunities [24].

The presence of children between 5 and 15 years in the household was significantly associated with episodes of diarrhoea. Since the diarrhoeal rate in this age group was similar to the rate for adults, it is likely that having a child of school-going age in the household is a risk factor for others in the household as these children may act as vectors. However, this is not previously reported in the literature and requires further investigation. Having a flush toilet in the house (as compared to in the yard) and inadequate handwashing were also associated with diarrhoea (although only marginally significant). Poor handwashing is a known risk factor for diarrhoeal diseases [7]; however, the association between diarrhoea and the location of the flush toilet requires further investigation and may be due to an unmeasured confounder. Food safety practice and food purchasing behaviour were not associated with diarrhoea in the current study.

Diarrhoeal episodes were relatively mild in the surveyed population, with a median duration of 2 days. The most common accompanying symptoms were abdominal cramps, headache, loss of appetite and fever. This is in agreement with systematic review data from low- and middle-income countries [25]. Only 21% of cases required healthcare intervention, which is similar to estimates of 21% from the United States [15]. Data from low- and middle-income countries estimate that 35.2% of diarrhoeal cases in children < 5 years [25] present to healthcare, but there are no data for rates in other age groups, which are expected to be lower. In the present study, of those that sought healthcare, the majority went to public clinics (61%), followed by pharmacies (22%) and public hospitals (17%). This differs to a community-based study in children < 5 years from the same community which found that 70% of cases sought healthcare at public clinics, 10% at a private practitioners, 10% at pharmacies and 5% at public hospitals [13]. This difference is probably because adults are more likely to seek healthcare at a pharmacy, rather than a public clinic, public hospital or private practitioner. This study was not powered to determine the difference in type of healthcare sought between age groups, however, no cases in children < 5 years sought healthcare at pharmacies. It is likely that diarrhoeal cases in children < 5 years were more severe, and that there was a lower threshold for seeking healthcare at other facilities. The number of individuals seeking healthcare for their illness underrepresents the subjective severity of illness, since 33% (12/36) of those who felt they needed healthcare were unable to access it. Many of the barriers to accessing healthcare identified here were also highlighted in the previous Soweto study, including issues with the health system (such as deficiencies in healthcare delivery, dissatisfaction with services, medications being out of stock) and personal reasons (such as time, finance and transportation constraints) [13].

The data presented here shows that only 4.6% of diarrhoeal cases in the community would have been detected through hospital surveillance and 16.1% through clinic surveillance. Analysis of routine diagnostic laboratory data would represent only 3.4% of the cases, being limited to those that had stool specimens collected. These findings are important to consider when interpreting the representativeness of such data.

We found community ORS knowledge to be poor in the surveyed population. Women and respondents with children in the household were more likely to have some knowledge of ORS, indicating that this information is most likely disseminated through baby and childcare clinics, a finding which was also reported in rural Botswana [26]. There is a gap in information dissemination for men and households without children, which should be addressed.

This study was limited by the reliance on a single household member to answer questions on behalf of other household members. This may have introduced bias, as respondents were less likely to accurately respond to questions regarding diarrhoeal episodes experienced by others and may have inflated episodes experienced themselves. This method has been used in similar studies [3, 20] and was preferable to collecting data on respondents only, as this may have biased results towards those that stay at home during the day. The study could have been strengthened by interviewing all members of included households; however, this was impractical. We were also unable to investigate the association between HIV and diarrhoeal disease at an individual level as this data was unavailable and could not be requested from respondents for ethical reasons. Level of education is known to be associated with diarrhoea, however this was not investigated in the current study as this variable was missing for the majority of HDSS members. Our findings are generalizable to many similar communities in South Africa, but may not be applicable to rural settings where living conditions and health-seeking patterns are likely to differ substantially. Therefore, understanding of diarrhoeal diseases at a community level could be strengthened by expanding this study to a larger geographical area. Causal inference and predictors of diarrhoeal disease could not be determined due to the cross-sectional nature of the study.

Research on diarrhoeal disease focusses on children under the age of 5 years because this age group is particularly vulnerable to illness and are more likely to seek healthcare and therefore to be detected through healthcare and laboratory surveillance. Notably, this study shows that diarrhoeal rates in older age groups are high at a community level, but are missed through routine healthcare- or laboratory-based surveillance. It is therefore recommended that routine surveillance be extended to include public clinics and pharmacies. We recommend that handwashing practices in this community be further investigated in order to produce targeted health messaging. We also recommend that health education on ORS as a low cost, effective intervention for diarrhoeal diseases should be made widely available, and include men and households without young children as target groups for such health education.

## Supplementary Information

**Additional file 1: Figure S1.** Health seeking for reported diarrhoeal episodes. **Table S1.** Factors associated with ORS knowledge.

## Data Availability

The datasets used during the current study is available from the corresponding author on reasonable request.

## Abbreviations

CHAMPS: Child Health and Mortality Prevention Surveillance network
CI: Confidence interval
GBD: Global Burden of Disease
HDSS: Health and demographic surveillance site
IQR: Interquartile range
IRR: Incidence rate ratio
IWI: International Wealth Index
ORS: Oral rehydration solution/salts
PY: Person-years
WHO: World Health Organisation
