# Supplementary material for: Diarrhoeal diseases in Soweto, South Africa, 2020: a cross-sectional community survey
Source: BMC Public Health. 2021 Jul 20;21:1431. doi: 10.1186/s12889-021-11470-9 (PMC8293521; doi:10.1186/s12889-021-11470-9)
Supplement: Supplementary file 1 — Additional file 1: Figure S1. Health seeking for reported diarrhoeal episodes. Table S1. Factors associated with ORS knowledge. [file 12889_2021_11470_MOESM1_ESM.docx]

**Diarrhoeal diseases in Soweto, South Africa, 2020: a cross-sectional community survey**

**Authors and affiliations**

Siobhan L. Johnstone^1,2^, Nicola A. Page^1,3^, Juno Thomas^1^, Shabir A. Madhi^4^, Portia Mutevedzi^4^, Nellie Myburgh^4^, Carlos Herrera^4^, Michelle J. Groome^4^

^1^Center for Enteric Diseases, National Institute for Communicable Diseases, Johannesburg, South Africa; ^2^School of Public Health, Faculty of Health Science, University of the Witwatersrand, Johannesburg, South Africa; ^3^Department of Medical Virology, Faculty of Health Sciences, University of Pretoria, Private Bag X323, Arcadia, 0007; ^4^South African Medical Research Council: Vaccines and Infectious Diseases Analytics Research Unit, Faculty of Health Sciences, University of the Witwatersrand, Johannesburg, South Africa

**Supplementary Tables and Figures**

Figure S1: Health seeking for reported diarrhoeal episodes

Table S1: Factors associated with ORS knowledge

|  | Some knowledge on ORS ^a^  N = 192 | | No knowledge on ORS  N = 182 | p-value |
| --- | --- | --- | --- | --- |
| Gender  Male (%)  Female (%) | | 29 (15.8)  154 (84.2) | 89 (51.1)  85 (48.9) | **<0.001** |
| Age in years (mean; IQR) | | 46.4 (44.1 – 48.7) | 44.5 (41.8 - 47.3) | 0.300 |
| Children less than 5 years old in the house  Yes  No | | 77 (40.1)  115 (59.9) | 50 (27.5)  132 (72.5) | **0.010** |
| Children between 5 and 15 years old in the house  Yes  No | | 104 (54.2)  88 (45.8) | 70 (38.5)  112 (61.5) | **0.002** |
| Wealth Index | | 84.7 (83.4 – 85.9) | 86.1 (84.7 – 87.5) | 0.132 |

^a^ Some knowledge on ORS defined as knowing the recipe or being able to name ingredients.
